# Expression of integrin β-7 is epigenetically enhanced in multiple myeloma subgroups with high-risk cytogenetics

**DOI:** 10.1186/s13148-023-01433-9

**Published:** 2023-02-04

**Authors:** Samrat Roy Choudhury, Stephanie D. Byrum, Duah Alkam, Cody Ashby, Fenghuang Zhan, Alan J. Tackett, Frits Van Rhee

**Affiliations:** 1 Pediatric Hematology-Oncology, Arkansas Children’s Research Institute, University of Arkansas for Medical Sciences, Little Rock, AR 72202 USA; 2 Department of Biochemistry and Molecular Biology, University of Arkansas for Medical Sciences, Little Rock, AR USA; 3 Myeloma Center, Department of Internal Medicine, University of Arkansas for Medical Sciences, Little Rock, AR 72205 USA; 4 Department of Biomedical Informatics, University of Arkansas for Medical Sciences, Little Rock, AR 72205 USA

**Keywords:** Multiple myeloma, *ITGB7*, T(14, 16) subgroup, DNA methylation, Super-enhancer, CRISPR

## Abstract

**Background:**

Oncogenic overexpression of integrin-β7 (*ITGB7*) in cases of high-risk multiple myeloma (MM) was reported to promote enhanced interactions between neoplastic plasma-B cells and stromal cells to develop cell-adhesion mediated drug resistance.

**Methods:**

Expression profiles of adhesion related genes were analyzed in a cohort of MM patients containing major *IgH* translocations or hyperdiploidies (HY), diagnosed at the premalignant monoclonal gammopathy of undetermined significance (MGUS; *n* = 103), smoldering multiple myeloma; (SMM; *n* = 190) or MM (MM; *n* = 53) stage. Differential expression was integrated with loci-specific alterations in DNA-methylation and chromatin marks in MM patients. A CRISPR-based targeted induction of DNA-methylation at the *ITGB7* super-enhancer (SE) in MM.1S cells was employed to intersect the impact of cis-regulatory elements on *ITGB7* expression.

**Results:**

*ITGB7* was significantly (*p* < 0.05) upregulated in patients with t(14;16) and t(14;20) subgroups in all MGUS, SMM and MM stages, but sporadically upregulated in t(4;14) subgroup at the MM stage. We demonstrate a predetermined enhancer state on *ITGB7* in primary-B cells that is maintained under bivalent chromatin, which undergoes a process of chromatin-state alterations and develops into an active enhancer in cases of the t(4;14) subgroup or SE in cases of the t(14;16) subgroup. We also demonstrate that while targeted induction of DNA-methylation at the *ITGB7*-SE further upregulated the gene, inhibition of *ITGB7*-SE-associated transcription factor bromodomain-4 downregulated expression of the gene.

**Conclusions:**

Our findings suggest an epigenetic regulation of oncogenic overexpression of *ITGB7* in MM cells, which could be critical in MM progression and an attractive therapeutic target.

**Supplementary Information:**

The online version contains supplementary material available at 10.1186/s13148-023-01433-9.

## Background

Multiple myeloma (MM) is a subtype of B-cell neoplasia, marked by abnormal clonal plasma cell infiltrations into the bone marrow (BM), that constitutes about 30% of all hematological malignancies [1]. The disease develops from asymptomatic monoclonal gammopathy of undetermined significance (MGUS), transitions through smoldering multiple myeloma (SMM), and ultimately transforms into MM. On the basis of global gene expression profiles (GEP), MM has been classified into 7 major molecular subgroups with overexpression of certain genes, such as *cMAF*, *MAFB*, *FGFR3*, *MMSET*, *CCND1*, *CCND2*, and *CCND3* [2, 3]. GEP-based classifications have been integrated with underlying genetic anomalies as detected with fluorescence in situ hybridization (FISH), including aneuploidy or *IgH* translocations, to stratify MM according to risk of poor outcome and to determine appropriate therapeutic interventions for patients [4–8]. For instance, high-risk subgroups of MM harbor high expression of *MMSET/FGFR3* (4p16), cMAF (16q23), or *MAFB* (20q12), while standard-risk is characterized by expression of *CCND1* (11q13) or *CCND3* (6p21) [9]. In contrast, the non-*IgH* translocation hyperdiploidy (HY) subgroups having overexpressed *CCND1* or *CCND2* involve a gene dosage mechanism from trisomic chromosomes, constitute almost half of the myeloma cases and have relatively favorable prognosis [10].

Phenotypic manifestation of underlying etiological genetic and epigenetic events often is associated with enhanced interactions between BM stromal cells and malignant plasma cells, which has been reported as a key contributor to MM pathogenesis [6, 11–14]. BM matrix is enriched with growth factors such as IL6 or chemokine proteins such as CXCL12, which facilitate networking between adhesion molecules and promote homing and invasion of MM cells into the BM and extracellular matrix (ECM) resulting in chemotherapeutic resistance [15–17]. Increased interactions between adhesion molecules of ECM (e.g., fibronectin, E-cadherin, or laminin) and integrins, heparin sulfate proteoglycans, or surface signal receptors on tumor cells results trigger anti-apoptotic properties of MM cells, leading to cell-adhesion–mediated drug resistance (CAM-DR) [18, 19]. The CAM-DR is particularly prevalent in patients with refractory MM, who have t(14;16) or t(14;20) translocations and innate resistance to proteasome inhibitors such as bortezomib, and show consistent with a grim prognosis [20–25].

The high-risk MM subgroup with the t(14;16) translocation accompanied by ectopic expression of cMAF has been reported to upregulate gene clusters that are involved in direct or indirect nodes of intersections with adhesion-related pathways [14]. For instance, cMAF–transactivated *CCND2* upregulates integrin β7 (ITGB7) and chemokine receptor CCR1 proteins to promote enhanced attachment of MM cells to stromal BM cells [18, 26, 27]. Marked upregulation of co-stimulatory molecules CD28 and its ligand CD86 on MM cells also were reported to contribute to CAM-DR in this subgroup [28]. While *ITGB7* expression regulated by the transcription factor cMAF and its function in BM adhesion have been relatively well illustrated in MM [18], recent reports have showed that epigenetic mechanisms play a critical role in the process of CAM-DR [12, 16]. For instance, class-I histone deacetylases sensitize MM cells to proteasome inhibitors, but inhibitors of the IGF-1R/PI3 K/Akt pathway reverse CAM-DR by promoting EZH2 dephosphorylation and H3K27 hypermethylation [29, 30]. However, the distribution of chromatin landscape on *ITGB7* and its putative cooperativity with underlying DNA methylation to promote malignant adhesion properties in high-risk MM remain undetermined.

In the present study, we demonstrate regulatory epigenetic networks (RENs) of oncogenic expression of adhesion molecules, particularly *ITGB7*, in patient-derived MM samples, including subgroups containing high-risk cytogenetics. We interrogated the functional RENs of *ITGB7* on a CRISPR (clustered regularly interspaced short palindromic repeat) platform and show that perturbation of at least one component of RENs impacts anomalous expression of the underlying gene and potentially controls MM cell proliferation.

## Methods

### Samples

BM aspirates were collected from patients with MGUS (*n* = 103), SMM (*n* = 190), or MM (*n* = 53) stages at diagnosis. The patients used in the study were selected based on the incidence of major *IgH* translocations, such as t(4;14), t(11;14), t(14;16), or t(14;20), and HY, as identified by FISH and/or next-generation sequencing/gene-expression profiling microarray. HY refers to the MM patients having amplification or gain (trisomy) of odd chromosomes (except for Chr.13). Because *CCND1* and *CCND2* are almost exclusively expressed in MM samples, the HY cases are further categorized as D1 (having overexpression of *CCND1*) or D2 (having overexpression of *CCND2*) subgroups [31]. MM cells were enriched by fluorescence-activated cell sorting (FACS) with over 98% CD138-positive cells (RoboSep; StemCell Technologies). Four pools (10 samples/pool) of plasma B cells isolated from healthy donors were used as a control. NCI-H929, U-266, MM.1S, and SACHI myeloma cell lines were included as representative lines with background for t(4;14), t(11;14), t(14;16), and t(14;20) translocation subgroups, respectively (Additional file 2: Table S1). Cell lines were purchased from the American Type Culture Collection (ATCC), except for SACHI (gifted by Dr. Valeriy Lyzogubov, Myeloma Center, UAMS). Cell lines were cultured in RPMI 1640 medium (Thermo Scientific) containing 10% heat-inactivated fetal calf serum (Hyclone Laboratories) in a humidified incubator (37 °C, 5% CO2). The demographic and clinicopathological characteristics of the patients are summarized in Additional file 2: Table S2.

### Gene expression profiling

Gene expression profiling was carried out on the Affymetrix U133 Plus 2.0 platform. CEL files were processed using R (v 4.2.0) with packages “affy” and “limma” [32]. An average GEP value for patient samples and cell lines was considered significant (for downstream analyses) if > onefold (log2) higher or lower than control plasma cells at *p* < 0.05.

### DNA-methylation sequencing

The enhanced reduced representation bisulfite sequencing (eRRBS) protocol was performed per our published guidelines [33]. Details of the method can be found in the Supplementary Information.

### ChIP sequencing and determining chromatin modifications

Chromatin immunoprecipitation (ChIP) and fixation were carried out as per manufacturer’s protocol (Active Motif Inc.) in MM.1S cells. Bromodomain-4 (BRD4), mediator-1 (MED1), H3K4me1 and me3, H3K27ac, H3K36me3, and H3K27me3 marks were investigated with MACS2 peak calling [6]. Chromatin marks represented for primary B cells and H929 cells or DNAse hypersensitivity (DHS) in MM.1S cells were downloaded from ENCODE dataset. Chromatin marks for U-266 cell line were downloaded from the BLUEPRINT epigenome database. The cMAF binding profile was predicted in reference to the human memory Th17 cells [34]. Accession ID of the chromatin marks, used in this study have been summarized in Additional file 2: Table S11.

### CRISPR targeting, pyrosequencing, and qPCR on *ITGB7* differentially methylated regions (DMRs)

A CRISPR-enabled fusion protein of dCas9 and DNMT3A was used to select 3 DMRs at the intragenic regions (body) of *ITGB7* for site-specific induction of DNA methylation. Additionally, 3 site-specific single guide RNAs (sgRNAs) were synthesized and co-transduced with dCas9-DNMT3A module in MM.1S cell line to increase loci-specific DNA methylation (Supplementary Sequences 1–3, Additional file 2: Table S12). MM.1S cells were transduced (LentiX, Takara) with the lentivirus packaging compatible dCas9-DNMT3A or sgRNA plasmids. Alterations in DNA methylation were identified with pyrosequencing (Pyromark 24 system: QIAGEN). Genomic DNA (approximately 600 ng) extracted from CRISPR-modified MM.1S cells was bisulfite converted, PCR amplified, and sequenced in triplicate (Additional file 2: Table S12). The possibility of DNA-methylation–induced changes in *ITGB7* expression was determined, in triplicate, with qPCR analysis (QuantStudio 6 and 7 system, SYBR green dye, primer sets described elsewhere) [18], relative to endogenous expression of *GAPDH*.

### BRD4 inhibition assay

1 × 10^4^ MM.1S cells, seeded per well in a 96-well plate were treated with JQ1, a BET (bromodomain and extra terminal) -BRD4 (bromodomain containing protein 4) inhibitor, at concentrations as low as 25 nM to 2 µM. We recorded cell viability (CellTiter-Glo; Promega) 24, 48, and 72 h after JQ1 treatment. Next, 2 × 10^6^ MM.1S cells were treated with 0.1 µM and 0.2 µM of JQ1 for determining the changes in protein expression of super-enhancer (SE)-related transcription factors (e.g., BRD4, MED1), histone mark H3K27ac, or ITGB7 using Western blots (WB) from whole-cell lysates after 24 h and 48 h of treatment. Considering JQ1 treatments induced cell death, we separated the live and dead cells in the culture using a dead cell removal kit (Miltenyi Biotec). Briefly, both JQ1 treated and untreated cells were mixed with microbeads, followed by separation through a LS-column in the magnetic field per manufacturer’s protocol. Live cells were lysed with RIPA buffer supplemented with protease inhibitor cocktail and phosphatase inhibitor. The concentration of cell lysates was determined with BCA protein assay kit (Pierce), and 20 ug of proteins were loaded on the 4–12% gradient gel (NuPAGE, Invitrogen). Antibodies that were used for WB in the study are listed in Additional file 2: Table S14.

### Statistical analysis

A 2-way ANOVA was used to determine significance in analysis of differential methylation. A non-parametric 2-tailed Mann–Whitney U test was performed for the rest of the analyses, and statistical significance was determined at *p* < 0.001 or < 0.05, as indicated.

## Results

### Adhesion-related genes are differentially expressed in molecular subgroups of MM across the stages of disease progression

**Fig. 1:**
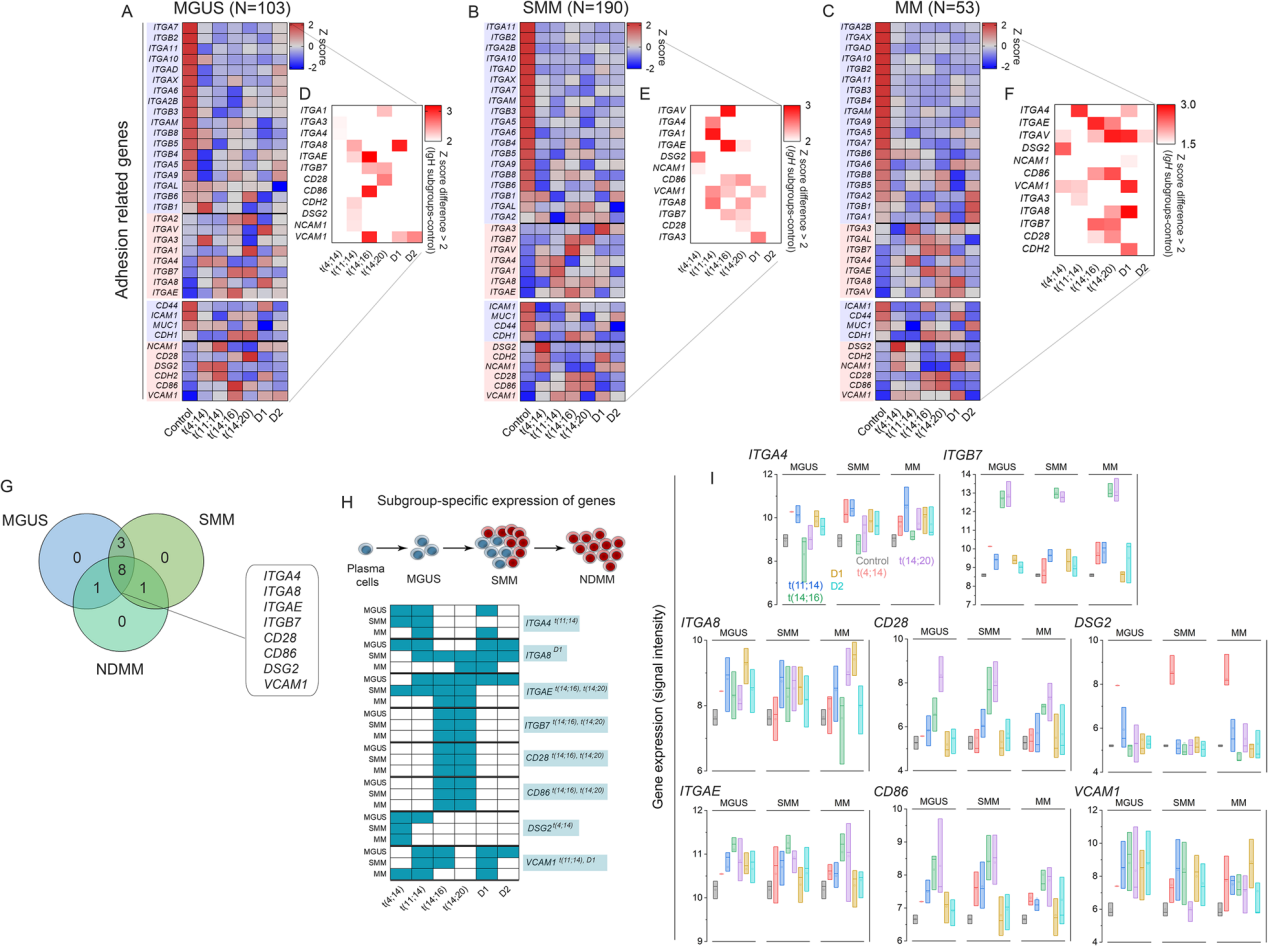
Heatmaps representing differential expression (z-score) of genes encoding adhesion-related integrins and non-integrins in t(4;14), t(11;14), t(14;16), and t(14;20) *IgH* translocation subgroups and D1 and D2 hyperdiploidy subgroups in patients diagnosed at (**A**) MGUS (monoclonal gammopathy of undetermined significance), **B** SMM (smoldering multiple myeloma), and **C** MM (multiple myeloma) stages. **D**–**F** Adjacent heatmaps were created with a subset of genes that have differential z-scores > 2 in MM subgroups versus control. **G** A Venn intersection diagram represents the genes that were consistently upregulated in all 3 stages of the disease. **H** Adhesion genes were filtered further, on the basis of their and consistent upregulation (Z score values) in certain MM subgroups at different stages. **I** The median differential expression (log2) of genes encoding 4 integrins (*ITGA4*, *ITGA8*, *ITGAE*, and *ITGB7*), 2 non-integrin proteins (*DSG2,* and *VCAM1)* and 2 co-stimulatory molecules (*CD28* and *CD86*) in patient samples of major *IgH* translocation and hyperdiploidy subgroups, represented as boxplots with interquartile range between 25 and 75%, compared to control

Earlier literature suggests that different adhesion molecules including integrin and non-integrin proteins are aberrantly expressed on the surface of neoplastic MM cells, which aid in the homing of MM cells to the BM stromal cells and develop CAM-DR (Additional file 2: Table S3). Additionally, dependency and co-expression of co-stimulatory molecules CD28 and CD86 with integrins, particularly *ITGB7*, has been reported in conjunction with MM pathogenesis [28]. We investigated differential expression of adhesion-related genes—18 candidates of α- and 8 candidates of β-subunits of integrins, 8 candidates of non-integrin adhesion molecules (*ICAM1*, *MUC1*, *CDH1*, *CDH2*, *CD44*, *DSG2*, *NCAM1* and *VCAM1*), and 2 co-stimulatory signaling molecules (*CD28* and *CD86*)—in samples from patients with initial diagnosis at MGUS (Additional file 2: Table S4), SMM (Additional file 2: Table S5), or MM (Additional file 2: Table S6) and representing 4 major *IgH* translocation and 2 HY subgroups [19, 35, 36]. Based on z-scores of expressions (log2), we categorized the integrin and non-integrin adhesion genes into 2 clusters in which expression in a disease subgroup was either downregulated or upregulated, relative to the control. Of the 26 genes encoding integrin adhesion molecules, 17 were mutually downregulated (boxed in blue, Fig. 1A–C) and 6 were mutually upregulated (boxed in red, Fig. 1A–C), relative to the controls, among all subgroups in MGUS, SMM, and MM stages. In contrast, of the 10 genes encoding non-integrin adhesion molecules, 4 were mutually downregulated (boxed in blue, Fig. 1A–C) and 6 were mutually upregulated (boxed in red, Fig. 1A–C) among the subgroups across the 3 stages of MGUS, SMM and MM.

Next, we narrowed gene number based on difference in z-scores (> 2) of expressions between individual subgroups and the control. Overall, the differentially expressed genes were upregulated (Fig. 1D–F) in the disease-subgroups, compared to control. Upon combining these gene-sets, we observed a panel of 8 genes (*ITGA4*, *ITGA8*, *ITGAE*, *ITGB7*, *CD28*, *CD86*, *DSG2,* and *VCAM1*) that were upregulated in at least one of the six subgroups in all 3 stages of the disease (Fig. 1G). Because the molecular subgroups have distinct underlying mechanisms of pathogenesis, we next investigated whether differential upregulation of each of the 8 genes was generic or specific to a subgroup [2]. Among the 8 genes, *ITGA4* was upregulated consistently in the t(11;14) subgroup, *ITGA8* in D1 subgroup, *DSG2* in t(4;14) subgroup, *VCAM1* in both t(11;14) and D1 subgroup*,* and *ITGAE*, *ITGB7*, *CD28*, and *CD86* were upregulated consistently in the MF cluster—i.e., the t(14;16) expressing high level of cMAF and t(14;20) subgroup expression high level of MAFB—in all 3 stages (Fig. 1H).

In the t(11;14) subgroup, *ITGA4* expression (median ± SD) was increased by 1.07-fold, compared to control (9.06 ± 0.44) in MGUS, 1.3-fold than control in SMM, and 1.5-fold than control in MM (Fig. 1I). *ITGB7* expression increased to 12.73 ± 0.52 in the t(14;16) subgroup and 12.78 ± 0.67 in the t(14;20) subgroup in MGUS, 12.93 ± 0.48 in the t(14;16) subgroup and 12.74 ± 0.37 in the t(14;20) subgroup in SMM, and 12.96 ± 0.46 in the t(14;16) subgroup and 12.85 ± 0.62 in the t(14;20) subgroup in MM, compared to control (8.60 ± 0.08) (Fig. 1I). Similarly, *ITGAE*, *CD28*, and *CD86* were consistently and significantly upregulated in the MF subgroups, compared to control across the stages of disease progression (Fig. 1I). In contrast, *ITGA8* and *VCAM1* were consistently upregulated in the D1 subgroup at all MGUS, SMM and MM samples, while *VCAM1* was also upregulated in t(11;14) subgroup at all 3 disease stages. *DSG2* was the only gene that were consistently upregulated in the t(4;14) subgroup across all three stages of the disease (Fig. 1I). Our results thus represent a subgroup-specific expression of adhesion-related genes that are upregulated from the earliest stage of MM.

### DNA methylation changes dynamically in adhesion-related genes in molecular subgroups of MM

**Fig. 2:**
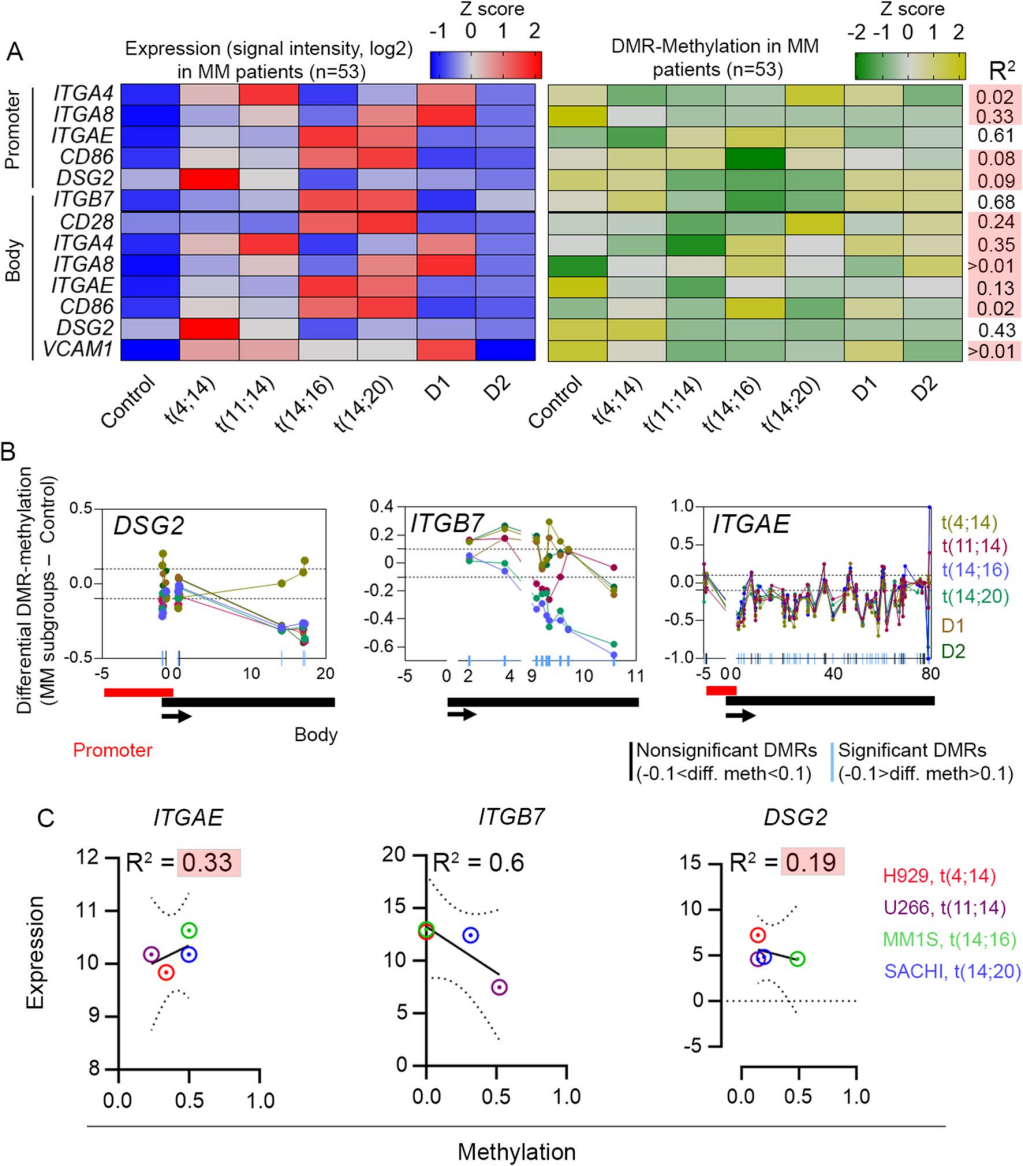
**A** Heatmaps representing correlation (Pearson’s coefficient, R^2^) between differential expression (z-score) and median DNA methylation (z-score) of genes encoding 4 integrins (*ITGA4*, *ITGA8, ITGB7*, and *ITGAE*), 3 non-integrins (*CDH2, DSG2, and VCAM1*) and 2 co-stimulatory signal transduction-associated molecules (*CD28*, and *CD86*) in patient samples (*N* = 53) of multiple myeloma (MM) of *IgH* translocation and hyperdiploidy subgroups versus control. **B** Distribution of DNA methylation along the differentially methylated regions (DMRs) in promoter or body of the differentially methylated and expressed genes in MM molecular subgroups. **C** A correlation analysis (R^2^ with 95% confidence band) between median DNA methylation and expression of the key adhesion-related genes in MM cell lines representing 4 major *IgH* translocations

Previously, we reported that DNA methylation tightly regulates gene expression in molecular subgroups of MM [6, 37]. Given consistent upregulation of genes encoding 4 integrins (*ITGA4*, *ITGA8,* *ITGAE*, and *ITGB7*), 2 non-integrins (*DSG2* and *VCAM1*), and 2 co-stimulatory signaling molecules (*CD28*, *CD86*) across developmental stages of the disease, we tested whether their expression levels are linked to changes in DNA methylation. Heatmaps of z-scores of median DNA methylation of the differentially methylated regions (DMRs) and heatmaps of z-scores of expressions of the corresponding genes in MM patients (*n* = 53) were plotted to evaluate correlations (R^2^) between methylation and expression (Fig. 2A). Based on our cut-off for the significance in correlation coefficient (R^2^ > 0.4), we found a strong correlation between methylation and expression of *ITGAE* (R^2^ = 0.61), *DSG2* (R^2^ = 0.43), and *ITGB7* (R^2^ = 0.68). The highest expression of *DSG2* in the t(4;14) subgroup was well matched to the high methylation level of the body-residing DMRs, whereas the reduced levels of gene expression in the remaining subgroups correlated with low levels of methylation at the body. In contrast, we observed atypical methylation–expression relationships for *ITGAE* and *ITGB7*. For instance, expression of *ITGAE* was highest in the t(14;16) and t(14;20) subgroups of MM, although methylation density at the promoter-residing DMRs also was highest. In contrast, expression of *ITGB7* was highest in the t(14;16) and t(14;20) subgroups, despite very low levels of methylation at the body-residing DMRs.

Next, we mapped the distribution of DMRs across the promoters and bodies of the 3 adhesion related genes. We identified 9 DMRs (72 CpG sites) in *DSG2*, including 4 (DMR-1 to -4) at the promoter and 5 (DMR-5 to -9) at the body (Fig. 2B, Additional file 2: Table S7). The median methylation of DMRs in the gene body of *DSG2* in t(4;14) subgroup was 0.45%, which was closest to the healthy donors (0.44%), but considerably higher than the remaining MM subgroups, where the median methylation did not exceed 20%. Noteworthy, the highest level of methylation was observed at the DMR-9 (84%), followed at DMR-8 (72%) and at DMR-7 (45%). The remaining body residing DMRs (DMR-5 and -6) were almost depleted of DNA-methylation (> 1%). Therefore, methylation density at DMR-7 to -9 in *DSG2* body may be linked to the high expression of the gene in the t(4;14) subgroup.

We mapped 9 DMRs (62 CpG sites) at the body of *ITGB7* (Fig. 2B, Additional file 2: Table S7). Except for DMR-8 and -9, DM (differential methylation > 10%, compared to control) was highly (> 20%) reduced across the DMRs in t(14;16) and t(14;20) subgroups. In contrast, DM across DMRs in t(4;14) and t(11;14) subgroups was quite variable, such that methylation was increased (DM > 10%) in 4 of 9 DMRs in the t(4;14) subgroup but decreased in 4 of 9 DMRs in the t(11;14) subgroup. In contrast, while we observed increase in DM in 3 DMRs (DMR-4, DMR-8, and DMR-9) in D1, 3 DMRs (DMR-7, DMR-8 and DMR-9) in D2 subgroup, a decrease in DM was at the DMR-1 in both the HY subgroups. *ITGB7* methylation alone, thus, cannot adequately explain the observed changes in gene expression. Given the strong correlation between methylation of promoter residing DMRs and expression in *ITGAE* (Fig. 2B, Additional file 2: Table S7), we then investigated the 3 DMRs (12 CpG sites) spanning the gene promoter. DM at most of these DMRs was increased, except at DMR-76 in the t(4;14) subgroup and at DMR-77 in t(14;20) in t(14;20) subgroups, where methylation was less than the control. Nevertheless, overall DM across the promoter residing DMRs in *ITGAE* cannot explain the differential expression in the molecular subgroups of MM samples.

Finally, we investigated the methylation distribution of adhesion-related genes in MM cell lines containing different *IgH* translocations. The total number of DMRs per gene in patient samples and MM cell lines were consistent, except in the case of *ITGB7* and *ITGAE*. In *ITGB7*, 2 DMRs were identified in MM cell lines, but 9 DMRs were identified in patient samples; in *ITGAE*, 76 DMRs were identified in MM cell lines, but 77 DMRs were identified in patient samples. However, the degree of DM and correlation between methylation and expression between MM cell lines and patient samples, were slightly different. Nonetheless, we observed a strong correlation (R^2^ = 0.6) between methylation and expression in *ITGB7* in MM cell lines (Fig. 2C, Additional file 2: Tables S8, S9).

### Changes in DNA methylation and chromatin state impact ITGB7 expression in specific subgroups of MM

**Fig. 3:**
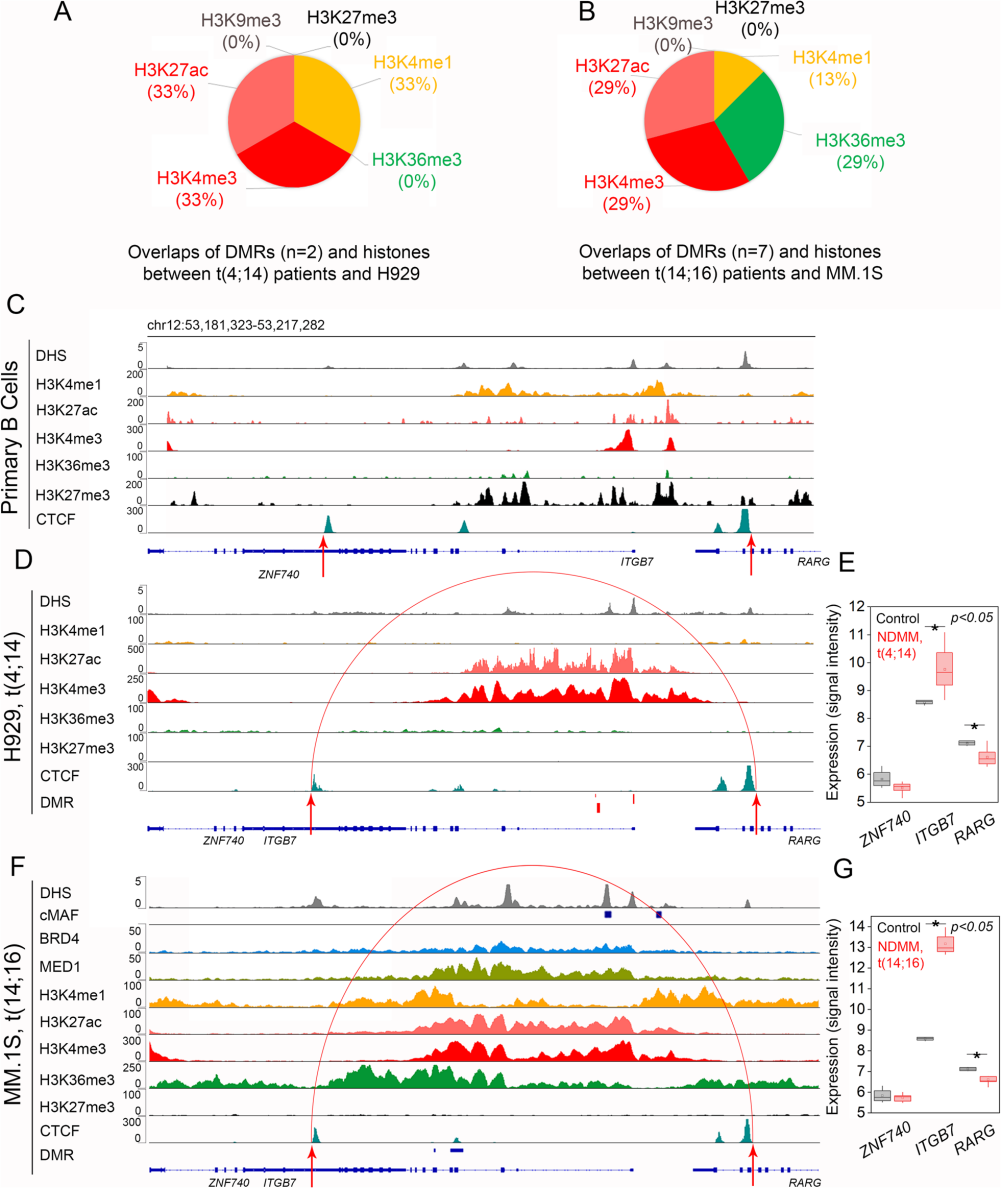
Pie charts representing percentage-distribution of overlapping regions (> 50 bp) between differentially methylated regions (DMRs) and histone enrichment (signal *p* > 50) mapped across the intragenic region of *ITGB7*. **A** *ITGB7* DMRs (*N* = 2) in patients with t(4;14) translocation overlapped with histone marks in H929 cell line. **B** *ITGB7* DMRs (*N* = 7) in patients with t(14;16) translocation overlapped with histone marks in MM.1S cell line. Chromatin-immunoprecipitation sequencing (ChIP-seq) data representing distribution and binding intensity of chromatin and transcription factors (TFs) at a region ± 10 kb upstream of the transcriptional start site (TSS) or up to the the 3’ end of *ITGB7* in (**C**) primary B cells, compared to (**D**) H929 cells (representing t(4;14) translocation) and to (**F**) MM.1S cells (representing t(14;16) translocation). Differential upregulation (*p* < 0.05) of *ITGB7* in both (**E**) t(14;14) subgroup and (**G**) t(14;16) subgroup, represented as a box chart with interquartile range between 25 and 75%, compared to control

Due to the tightly linked association between DNA methylation and histones in cancer, including MM [6, 38], we examined, if overlaps (≥ 50 bp) between DMRs in MM patient subgroups to histone marks in representative MM cell lines could predict cis-regulatory regions of the adhesion genes. We captured and presented loci enrichment of 4 activating histones (H3K4me1, H3K4me3, H3K27ac, and H3K36me3) and 1 inactivating histone (H3K27me3). Among the 8 adhesion-related genes, only *ITGB7* showed adequately overlapping DMRs and histones; this was particularly observed in H929 [t(4;14) subgroup] and MM.1S [t(14;16) subgroup] but not in U-266 [t(11;14) subgroup] (Additional file 2: Table S10). In the t(4;14) subgroup, we identified 2 (4 CpG sites) out of 9 DMRs at the *ITGB7* intragenic region (i.e., body), where methylation was increased by 22% and 17%, (*p* < 0.05), respectively, and coincides equally (33%) with histone marks H3K4me1, H3K4me3, and H3K27ac (Fig. 3A). In contrast, in the t(14;16) subgroup, we identified 3 DMRs in which methylation (median% ± SD) decreased by 50% ± 0.08 and was coincident (13%) with H3K4me1. We also identified 7 (58 CpG sites) out of 9 DMRs in the t(14;16) subgroup in which methylation decreased by 30% ± 0.13 and was coincident equally (29%) with histone marks H3K4me3, H3K27ac, and H3K36me3 (Fig. 3B).

Upon noting that *ITGB7* has adequate overlap between differential DNA methylation and histones, we further examined its chromatin state transitions at a region spanning 10 kb upstream of the transcription start site (TSS) to 10 kb downstream of the 3’ end of the gene in H929 cells and MM1S cells, compared to primary B cells (Additional file 2: Table S11). We identified a 23-kb window—from 6 kb upstream of TSS to 3 kb downstream of the 3’-end—that was marked by CTCF (CCCTC-binding factor) TFs bound at the edges. In primary B cells, we noticed characteristics of bivalent chromatin on *ITGB7*, such that co-occupancy with H3K27me3 and H3K4me1 marks was observed at the promoter (1.4 kb region, 855 bp upstream to TSS) and intragenic regions (intron, exons 1–3, and exon 5) of the gene (Fig. 3C). In contrast, in H929 cells (t(4;14) subgroup), we observed a broad domain (12 kb) co-occupied by H3K4me3 and H3K27ac (Fig. 3D). This de novo epigenetic broad domain also coincided with 2 DMRs containing an intermediate level of DNA methylation (40% ± 0.2) that is 20% higher than the control. Thus, the REN on *ITGB7* in the t(4;14) subgroup reflects an example of an active enhancer, which could explain the increased expression (onefold, log2; *p* < 0.05) of the gene in this subgroup, compared to control (Fig. 3E). The most dramatic changes in the chromatin landscape and DNA methylation of *ITGB7* were observed in MM.1S cells, where acquired enrichment in H3K4me1 was noted in addition to H3K4me3 and H3K27ac (Fig. 3F). Appearance of H3K4me1 also is concomitant with the major drop in DNA methylation across 7 DMRs in t(14;16), relative to control. This epigenetic architecture is favorable for creating an open chromatin state, as marked with increased DHS and binding intensities of SE-associated TFs, such as BRD4 and MED1. Interestingly, we also observed an increase in intensity of H3K36me3 binding, overlapping the activating SE marks and DMRs. We also observed 2 MAF binding sites, one at the gene’s promoter and one adjacent to its TSS. Overall, the architecture of REN on *ITGB7* represents formation of an SE-assembly, which allows significant upregulation (4.3-fold, log2; *p* < 0.05) of the gene’s expression in the t(14;16) subgroup, relative to control (Fig. 3G).

*ITGB7* thus represents a classic example of chromatin state transition in MM; in B cells, the gene is under H3K27me3 repression, but it acquires an active enhancer state in the t(4;14) subgroup and a SE state in the t(14;16) subgroup. The epigenetic enhancement of expression of *ITGB7* seems particularly critical to development of the disease, because expression of the genes neighboring *ITGB7* (i.e., *ZNF740* or *RARG*) remain unaltered, compared to control, in the subgroups from samples of MM (Fig. 3E, 3G). Enhancement of *ITGB7* expression is also deemed to be subgroup specific, given there is no changes observed in chromatin state in U-266 cells, representing t(11;14) subgroup (Additional file 1: Fig. S5).

### Induction of DNA methylation at *ITGB7* intragenic enhancer further increases its expression

**Fig. 4:**
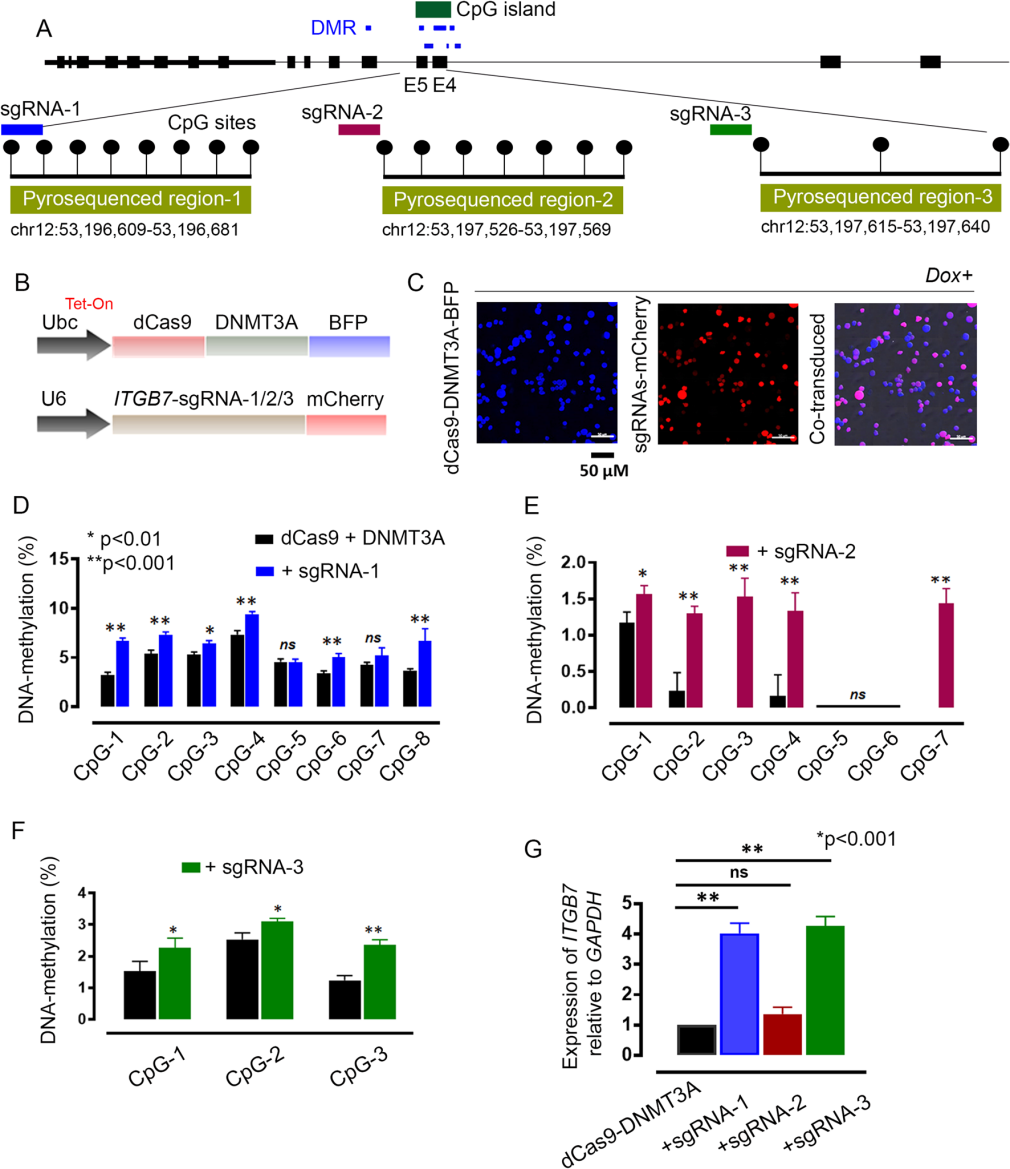
**A** Three differentially methylated regions (DMRs) spanning exon 4 to exon 6 of *ITGB7* were selected for targeted induction of DNA methylation, on the basis of their epigenetic regulatory roles in gene expression. **B** Targeted DNA methylation was aimed by designing a fusion protein of deactivated cas9-endonuclease (dCas9) and catalytic domain of DNA-methyltransferase 3A (DNMT3A-CD), and *ITGB7*-DMR targeting single guide RNAs (sgRNAs). **C** MM.1S cell line [t(14;16) translocation] was co-transduced with dCas9-DNMT3ACD and a combination of sgRNAs in the presence and absence of doxycycline for induction. Cells transduced with individual constructs or dual constructs were sorted with flow cytometry, based on the tagged fluorophore. **D**–**F** We performed region-specific pyrosequencing to determine changes (*p* < 0.01 or *p* < 0.001) in DNA methylation per CpG site at DMR-1, -2 and -3 before and after induction of dCas9-DNMT3ACD plus sgRNAs. **G** The impact of targeted changes in DNA methylation on *ITGB7* expression was determined with qPCR

We used a CRISPR tool to interrogate the enhancer regulation of *ITGB7* by targeted induction of DNA methylation at the selected DMRs, which are part of a CpG island (spanning exon 4 to exon 6) and overlap the region with abundant SE-associated chromatin marks (Fig. 4A). We designed and developed a doxycycline (Dox)-inducible (Tet-on) fusion construct that contains deactivated cas9 endonuclease (dCas9) and the catalytic domain of DNMT3A (DNMT3ACD), in addition to 3 independent sgRNAs (Fig. 4B) that are complimentary to the targeted DMRs in *ITGB7* (Additional file 2: Table S12, Supplementary Sequences 1–3). MM.1S cells were co-transduced with dCas9-DNMT3A and different combinations of sgRNA in the presence or absence of Dox and then sorted (≥ 80%) based on tagged blue fluorescent protein and mCherry on dCas9-DNMT3A or sgRNA constructs, respectively (Fig. 4C, Additional file 1: S4).

The treated cells were profiled for changes in DNA methylation by using pyrosequencing (Additional file 2: Table S13). We observed significantly increased (*p* < 0.001) DNA methylation in 6 of 8 CpG sites of DMR-1 in Dox-induced and co-transduced cells (Fig. 4D). Similarly, increased methylation was observed in 4 of 6 CpG sites in DMR-2 (Fig. 4E) and at all 3 CpG sites in DMR-3 (Fig. 4F). The impact of induction on DNA methylation was demonstrated by alterations in gene expression. Cells treated with the combination of dCas9 + DNMT3A and sgRNA-3, compared to those treated with the dCas9 fusion protein, had 3.2-fold (*p* < 0.001) more gene expression. A similar increase (threefold) in gene expression was observed for cells treated with the combination of dCas9 + DNMT3A and sgRNA-1. In contrast, no significant changes in gene expression were observed in cells treated with dCas9-DNMT3A and sgRNA-2 (Fig. 4G). Notably, we did not perceive noticeable changes in gene expression in the absence of Dox induction (Additional file 1: Fig. S6).

In summary, our results demonstrate that DNA methylation might play a critical role in the function of REN on *ITGB7*. We demonstrated that, while a low level of methylation is favorable for forming an open chromatin state and for assembly of SE-associated proteins, induction of DNA methylation might further increase the gene expression, possibly through crosstalk with the overlapping intragenic H3K36me3 mark.

### Inhibition of BRD4 suppresses *ITGB7* expression and myeloma cells proliferation

**Fig. 5:**
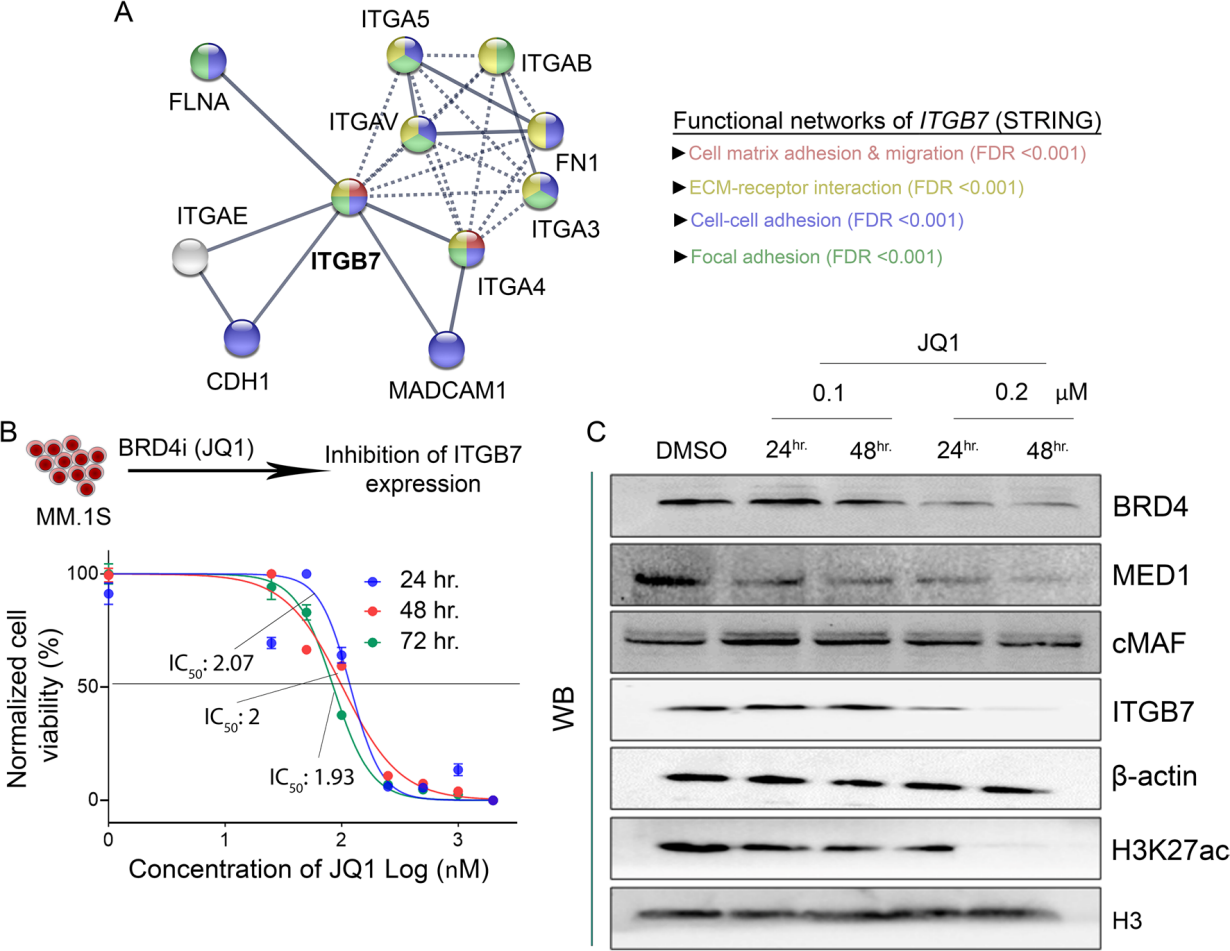
**A** A predictive interactome map of *ITGB7* was constructed with STRING protein network analysis (confidence ≥ 9.0), which showed strong involvement (FDR < 0.001) of the protein in functions such as cell matrix adhesion and migration, extracellular matrix interactions, cell–cell interactions, and focal adhesion. **B** MM.1S cells were treated with a bromodomain-4 (BRD4) inhibitory drug, JQ1, at concentrations ranging from 25 nM to 2 µM; half-life of inhibitory concentration (IC_50_) was recorded after 24 h, 48 h, and 72 h of incubation. **C** JQ1 treatment at 0.1 µM (IC_50_ at 24 h) and 0.2 µM (onefold higher than IC_50_ at 24 h) effectively reduced expression of super-enhancer–bound epigenetic marks, such as BRD4, MED1, or H3K27ac, with simultaneous reduction in expression of underlying genes, as exemplified with *ITGB7*. In contrast, expression of cMAF or endogenous controls (β-actin, H3) remained unaltered

*ITGB7* is part of an integrin family and is key in interactions with cadherins and receptor molecules of ECM, which play a critical role in the pathogenesis of MM and other cancers [18, 39] (Fig. 5A). Based on our cumulative findings, we recognized that *ITGB7* expression is epigenetically enhanced in the high-risk cytogenetic subgroups in MM. In particular, in t(14;16) subgroup, the gene is under SE control and is enriched for SE-associated TFs. Given the propensity of BRDs to bind acetylated lysines in histone tails, and their co-occupancy at open chromatin (as evident with the DHS signal) peaks, we used an inhibitor of BRD4 (BRD4i) to suppress the expression of *ITGB7*. JQ1, a BET-BRD4i has previously been shown to possess anti-proliferative and anti-apoptotic effects against the MM cells [40] We treated MM.1S cells with JQ1, at concentrations from 25 nM to 2 µM and determined cell viability at 24, 48, and 72 h. We found dose-dependent and time-dependent decreased viability in response to JQ1 treatment: the half maximal inhibitory concentration (IC_50_) changed from 0.11 µM at 24 h., to 0.1 µM at 48 h., to 0.08 µM at 72 h. (Fig. 5B). We further treated the MM.1S cells with JQ1 with 0.1 µM (IC_50_ of 48 h.) and with onefold higher than (0.2 µM) IC_50_ and determined effects on expression of SE-associated chromatin and TFs. We observed that JQ1 treatment for 48 h. completely diminished the BRD4, MED1, and H3K27ac expression (Fig. 5C). BRD4 inhibition also was accompanied by a significant reduction of *ITGB7* expression. Noteworthy, BRD4 inhibition had no effects on the endogenous expression of cMAF (Fig. 5D). Our collective data support the coincidence and functional dependence of *ITGB7* on the SE-associated chromatin marks, which is supported by the simultaneous inhibition of both SE marks and expression of *ITGB7* with BRD4i treatment.

## Discussion

After the IgH class switching mechanism, MM cells start releasing an array of adhesion molecules that mediate enhanced intercellular interactions between MM cells and stromal cells, and they also transduce signals for extracellular interactions with ECM components [14, 18]. These enhanced cellular interactions are imperative for MM cell survival and for the development of CAM-DR and osteolytic lesions [41]. Integrins, part of an extensive family of glycoproteins, have been identified as the key players of the BM–ECM interactions in MM [14]. In particular, *ITGB7* is an integrin family member that is exceptionally upregulated in 2 molecular subgroups of high-risk MM—the MS subgroup [t(4;14)] and MF subgroups [t(14;16) and t(14;20)]. Conditional knockdown of *ITGB7* results in reduced cell viability and renders MM cells sensitive to proteasome inhibitors [18].

Based on major *IgH* and HY subgroups, we observed a propensity for downregulation (65%) rather than upregulation (23%) among the genes encoding integrins. Of the upregulated genes, *ITGA4* (α4) was consistently and particularly overexpressed in the t(11;14) subgroup. In contrast, *VCAM1* was significantly upregulated in both the t(11;14) and D1 subgroups. We also noticed specific upregulation of *DSG2* in all 3 stages of the disease in the t(4;14) subgroup. *ITGAE* (αE) and *ITGB7* (β7) were selectively upregulated in the MF cluster. *ITGAE* was also differentially upregulated in the t(11;14), D1 and D2 subgroups at MGUS or in t(4;14) and t(11;14) subgroups in SMM stages, but expression of the gene was absent altogether at the MM stage. Similarly, *VCAM1*, which encodes the ligand of *ITGB*7, was differentially upregulated in the t(14;16) subgroup at MGUS and SMM stages, but its upregulation did not continue in the MM stage. We also investigated expression of two co-stimulatory signal molecules CD28 and CD86 that were reported to be critical in MM cell survival [28]. Silencing of either of these signaling molecules were found to downregulate *ITGB7* mRNA expression and is assumed to have impact in cellular adhesion in the disease. Both CD28 and CD86 were upregulated in MF subgroups at all 3 stages of the disease, but this was not significantly (*p* < 0.05) correlated with the epigenetic regulators such as DNA-methylation.

In the current study, we demonstrated that alterations in DNA methylation of adhesion-related genes among molecular subgroups of MM samples are dynamic and do not necessarily impact gene expression via canonical methylation–expression relationships. This warrants close investigation, on a gene-by-gene basis, into the co-incidence and dependency of DNA methylation with other epigenetic regulators in regulation of expression. For instance, the oncogenic expression of cMAF was reported to regulate *ITGB7* function, particularly in the t(14;16) subgroup of MM, but the mechanism of *ITGB7* upregulation in the other molecular subgroups of MM is not fully understood [42]. Herein, we show that *ITGB7* is repressed due to concentrated H3K27me3 marks at the gene’s TSS and upstream promoter in primary B cells. In contrast, a dynamic interplay between chromatin marks and DNA methylation was observed in MM subgroups. In the t(4;14) subgroup, the H3K27me3 repressive chromatin mark was replaced by H3K4me3 and H3K27ac activating marks. This observation is well aligned with the previous literature, where MMSET in t(4;14) subgroup has been reported to induce global demethylation of activating H3K36, while reducing the trimethylation of repressive H3K27 [43, 44] De novo enrichment of the activating histone marks in the t(4;14) subgroup was supported by an intermediate level of DNA methylation at the overlapping DMRs (Fig. 3D); however, intensities in the DHS signal in H929 cells were not significantly different from those of primary B cells. Thus, the REN suggests a functional broad epigenetic enhancer domain on *ITGB7* in the t(4;14) subgroup [45]. In contrast, exceptional upregulation of *ITGB7* in the t(14;16) subgroup is supported by dramatic changes in the gene’s chromatin landscape. Severe depletion of DNA methylation levels at the enhancer related intragenic DMRs were consistent with the opening in chromatin (increased DHS intensity) and the enrichment in activating histones and TFs (Fig. 3F). The CTCF-marked region (23 kb), spanning the upstream promoter and most of the intragenic regions of *ITGB7*, could be considered an activation loop that selectively increases the gene’s expression and leaves the adjacent genes (i.e., those outside the loop) unaltered.

We showed a unique operational SE network on *ITGB7* intragenic DMRs in the t(14;16) subgroup in which H3K36me3 coincides with H3K4me1, H3K4me3, and H3K27ac. A similar REN was reported as a subclass of active enhancers in mouse embryonic stem cells [46]. Considering the tight association between the enhancer related DMRs and chromatin marks on *ITGB7*, we aimed to target specific alterations in DNA methylation at these loci. For the 3 enhancer-related DMRs chosen for the site-directed alterations in DNA methylation in MM.1S cells, where methylation levels were consistent with those of patient-derived samples of the t(14;16) subgroup (Additional file 1: Fig. S1–S3). Targeted induction of DNA methylation at the enhancer-related DMRs indicated a further increase of gene expression (Fig. 4D–G). However, there observed a disparity between the changes in DNA-methylation and expression in the target regions. For instance, we observed no changes in gene expression despite significant increase in DNA-methylation in 5 (CpG-1, CpG-2, CpG-3, CpG-4, and CpG-7) out 7 CpG sites at the region, targeted by sgRNA-2 (Fig. 4E). Lack of changes in methylation at the CpG-5 and CpG-6 could be partly due to the fact that the target CpG-sites are already masked or occupied with endogenous TFs, leaving a relatively narrow window of DNA sequence available for binding sgRNAs. There is also a possibility that methylation at CpG-5 or CpG-6 harbors the epigenetic switch that can regulate the changes in expression. Nonetheless, the increased gene expression in conjunction with the changes in DNA methylation in region-1 and region-3, could be ascribed to possible crosstalk with H3K36me3, which may aid elongation during transcription of the gene.

Considering the presence of functional dependency of epigenetic regulators in the REN of *ITGB7*, we showed that perturbation of BRD4 in these RENs has an effect on the underlying gene expression. Interestingly, our data show that, while BRD4i (JQ1) treatment can effectively mop up enhancer marks such as BRD4, MED1, and H3K27ac, it can also reduce *ITGB7* expression. This supports the concept that *ITGB7* expression is epigenetically enhanced in MM subgroups and could be altered by maneuvering the epigenetic modifications. Our results also showed that inhibition of BRD4 with JQ1 treatment did not interfere with the endogenous cMAF expression in MM.1S cells, which however do not nullify the importance of cMAF regulation of *ITGB7* expression. Previously, studies involving overexpression or dominant inhibition of cMAF has established the importance of this TF on *ITGB7* expression and cell adhesion in MM [25]. Additionally, a number of genes including *CCND2, CCR1, ITGB7* or *Notch* were found to be commonly regulated by cMAF and other MAF candidates such as MAFB in MM [47]. Therefore, binding of MAF candidates to the REN of *ITGB7* may synchronize with SE-associated proteins and chromatin marks that regulates oncogenic overexpression of the gene. Future studies should be aimed at designing target specific inhibitors of cMAF or SE-associated TFs at the *ITGB7* enhancer loci to gain further insight into the ITGB7 gene-regulation. Additionally, cell death induced by JQ1 at the concentrations used here may impact the degree of changes in the ITGB7 protein expression, since cells undergoing the process of apoptosis or necrosis could have significantly different gene expression, that are independent of the direct effects of this inhibitor on super-enhancers. Therefore, future studies are warranted to investigate whether BRD4i may be repurposed as therapeutic option for CAM-DR in MM. In conclusion, we present the architecture and functionalities of de novo RENs in *ITGB7* in the high-risk molecular subgroups of MM. Because *ITGB7* expression is epigenetically enhanced in MM, small-molecule inhibitors that target enhancer assemblies might show promise for controlling CAM-DR and overall malignant growth in MM.

## Supplementary Information

**Additional file 1**. The supplementary methods, sequences, and figures in this study.

**Additional file 2**. The supplementary tables in this study.

## Data Availability

The arrayed data have been provided as supplementary information. The genome-wide raw datasets used in the current study are available from the corresponding author and with permission of University of Arkansas for Medical Sciences on reasonable request.
